# Spatially and temporally resolved metabolome of the human oral cavity

**DOI:** 10.1016/j.isci.2024.108884

**Published:** 2024-01-12

**Authors:** Alessio Ciurli, Yassene Mohammed, Christine Ammon, Rico J.E. Derks, Damien Olivier-Jimenez, Quinten R. Ducarmon, Marije Slingerland, Jacques Neefjes, Martin Giera

**Affiliations:** 1 Oncode Institute and Cell and Chemical Biology, Leiden University Medical Center, 2333 ZA Leiden, the Netherlands; 2 Center for Proteomics and Metabolomics, Leiden University Medical Center, 2333 ZA Leiden, the Netherlands; 3 Structural and Computational Biology Unit, European Molecular Biology Laboratory, Heidelberg, Germany; 4 Department of Medical Microbiology, Leiden University Medical Center, 2333 ZA Leiden, the Netherlands; 5 Department of Medical Oncology, Leiden University Medical Center, 2333 ZA Leiden, the Netherlands

**Keywords:** Oral medicine, Biomolecules, Biological constraints, Metabolomics

## Abstract

Saliva is a complex bodily fluid composed of secretions by major and minor salivary glands. Salivary glands and their secretions are known to be unevenly distributed in the human oral cavity. Moreover, saliva flow rate and composition vary across locations and time of the day. This remarkable heterogeneity of salivary secretions suggests that different subtypes of saliva fulfill different functions. By coupling a non-invasive and facile collection method with comprehensive metabolomic profiling, we investigated the spatial and temporal distributions of salivary components. We identified location-specific metabolite profiles, novel oscillating metabolites, and location-specific diurnal patterns. In summary, our study paves the way for a deeper and more comprehensive understanding of the complex dynamics and functionalities of the salivary metabolome and its integration in multi-omics studies related to oral and systemic (patho-)physiology.

## Highlights

• Spatially and temporally resolved metabolome of the human oral cavity
• Identification of location-specific circadian oscillations
• Identification of location and time-specific metabolites

## Graphical abstract

**Figure:**
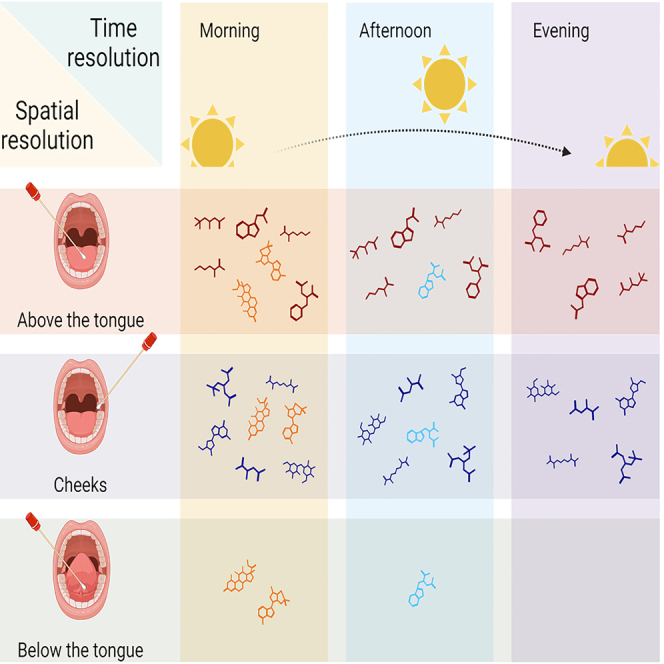

## Introduction

Saliva is an information-rich biofluid with regard to its diagnostic value and biochemical composition. Saliva covers a variety of functions such as lubrication, pH control, food particle clearance, maintenance of tooth integrity, antibacterial activity, taste, and digestion. Even more so, saliva is reflective of a subject’s hormonal, immunologic, and neurologic status, as well as nutritional metabolic influences,^1^ reflecting both oral and systemic metabolic alterations.^2^ In combination with its accessibility and facile, non-invasive collection, it has been proposed as a promising diagnostic fluid,^3^ as for example illustrated by the increasing number of studies now focusing on the development of easy-to-use sensors using saliva.^4^ Salivary samples can be used for monitoring a broad range of compounds, from endogenous metabolites (e.g., glucose) to therapeutic or recreational substances (e.g., caffeine) and hormones (e.g., cortisol and cortisone). In addition to major and minor gland secretions, there are other sources of metabolites including, the oral mucosal lining and oral microbiome, serum-derived filtrate fluid, as well as exogenous sources.^5^ The salivary secretions are rather complex and diverse. Within the major salivary glands, parotids produce a serous-type fluid that is released on the cheeks (CK) through the Stensen’s duct, whereas submandibular and sublingual glands excrete mucous-type fluids, which are both released on the mouth floor, or below the tongue (BT), through the Wharton’s and Bartholin’s ducts, respectively. Minor glands contributing to saliva production are found throughout the oral cavity; the palatal and palatoglossal secrete above the tongue (AT), the buccal and labial glands contribute to saliva in the CK, and the lingual glands release their fluids above and below the tongue depending on their exact location.^1^^,^^6^ The difference in the type of salivary secretions, e.g., serous- or mucous-type, reflects the variable compositions of specific salivary fluids^7^ suggesting that glands fulfill different functions and consequently can provide unique biological information. Indeed, as shown by us^8^ and others^9^ these spatial and functional differences are reflected in location-specific molecular characteristics and warrant spatially resolved approaches. In addition to its heterogeneous spatial distribution, saliva is also under circadian control, displaying daily variations in flow rate and composition.^6^^,^^10^ Salivary metabolites such as melatonin^11^^,^^12^^,^^13^ and cortisol^14^^,^^15^^,^^16^^,^^17^ are used as circadian biomarkers. The circadian rhythm regulates many aspects of mammalian physiology ranging from energy homeostasis to sleep to the detoxification of xenobiotics.^18^^,^^19^ While the circadian transcriptome and proteome have been mapped in detail^20^ the circadian metabolome is much less studied, even though circadian control has been reported.^21^^,^^22^ However, these studies neglected the spatial component which has been shown to significantly influence the metabolic composition of saliva.^8^^,^^9^ Given the chemical complexity of salivary secretions, it is plausible to hypothesize that the circadian rhythm affects salivary glands and controls the local saliva composition. Consequently, we simultaneously sampled saliva in a spatial and temporal fashion aimed to unveil the unique circadian regulations locally taking place within the human oral cavity. We, therefore, set out to sketch a spatially and temporally resolved metabolic map of the oral human cavity. We confirm the high degree of spatial specificity of the oral cavity through a dataset of structurally identified metabolites and present location-specific diurnal patterns as well as novel oscillating metabolites. Moreover, reflecting our findings, spatially and temporally collection of saliva should be considered the gold standard for any multi-omics integration within the oral domain. Finally, our study paves the way for a deeper understanding of the dynamics and functionalities of the salivary metabolome and its importance for molecular studies related to oral and systemic (patho-)physiology.

## Results

**Figure 1:**
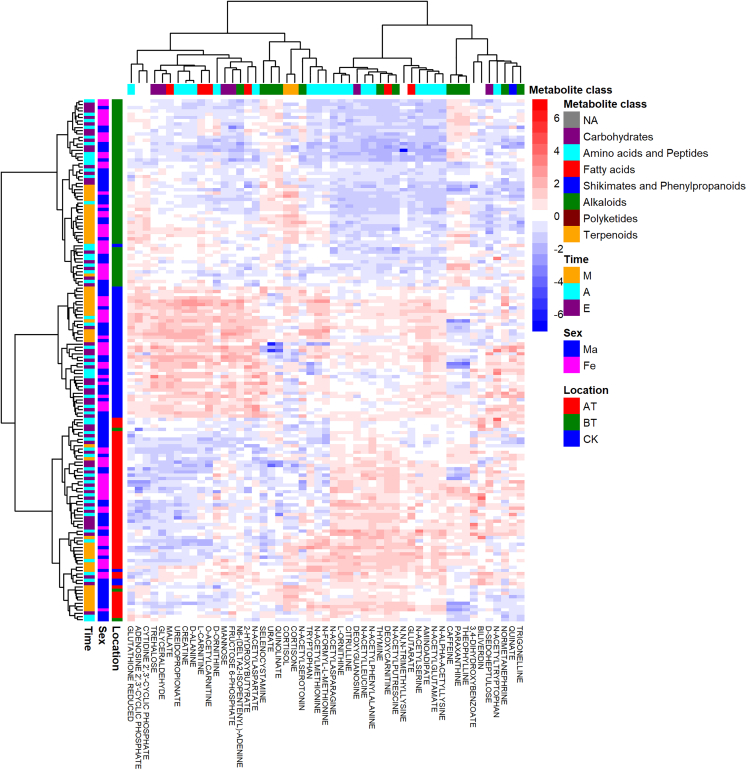
Distinctive metabolite heatmap across all analyzed samples Rows are samples and columns are metabolites. The heatmap shows the top ten discriminatory metabolites between groups. For the complete heatmap see supplementary materials Figure S1. Determined metabolite levels were log transformed and *Z* score normalized. Samples are annotated by time (M = morning, A = afternoon, E = evening), location (AT = above the tongue, BT = below the tongue, CK = cheek), and sex (Ma = male, Fe = female). Metabolites are annotated with molecular classification on the pathway level as determined by Natural Product Classifier.^25^

**Figure 2:**
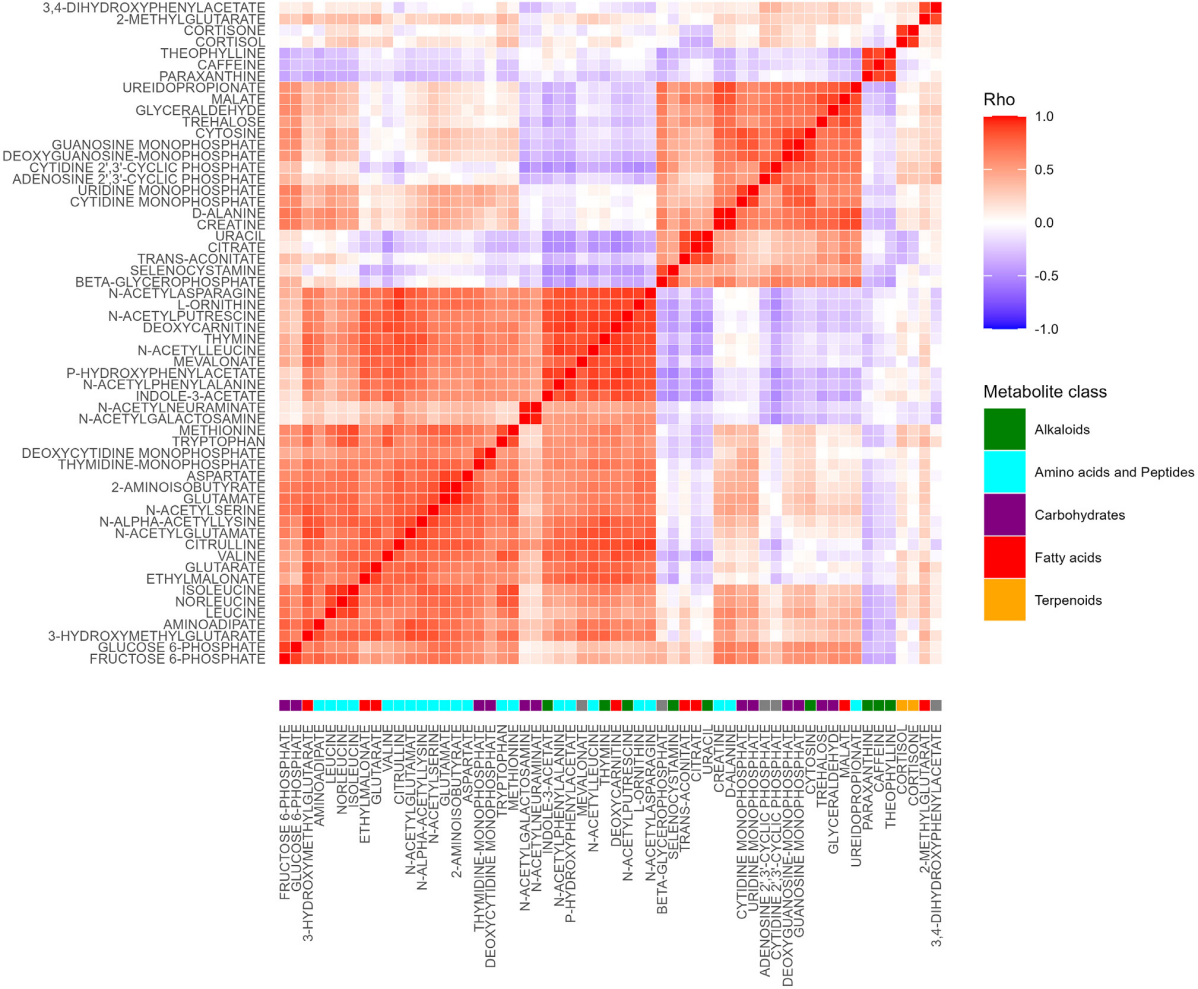
Spearman correlation matrix of highly correlated metabolites The correlation matrix was generated using Spearman’s rank correlation and, subsequently, filtered using a correlation coefficient cut-off of 0.8 (n = 159). Metabolites are annotated with molecular classification on the pathway level as determined by Natural Product Classifier.^25^ Prior correlation, data were log-transformed, and *Z* score normalized. For the complete correlation matrix see Figure S2.

**Figure 3:**
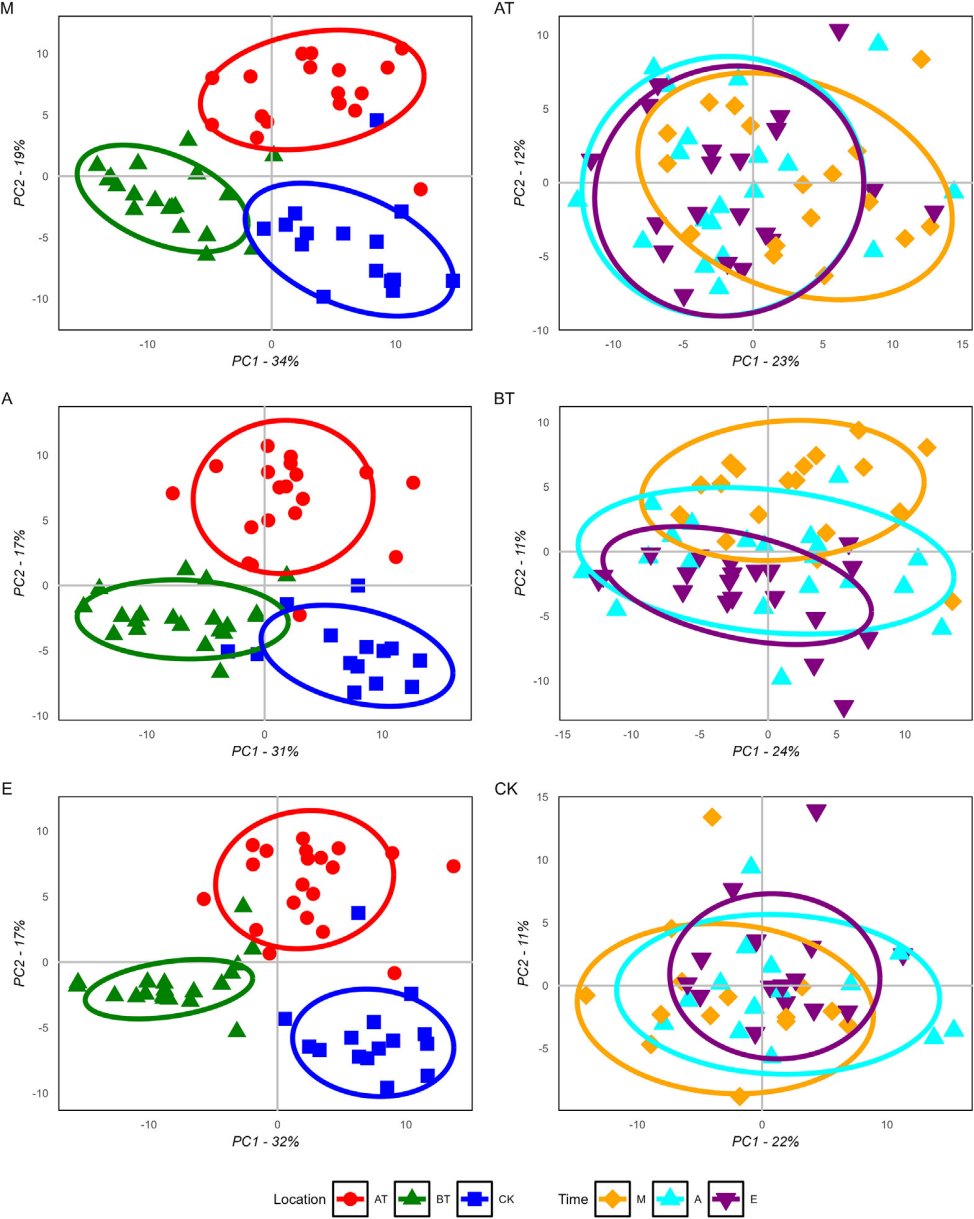
PCA subset score plots Prior to PCA computation, the full dataset was subset for location (AT = above the tongue, BT = below the tongue, CK = cheek) and time (M = morning, A = afternoon, E = evening) and subsequently log-transformed, and *Z* score normalized. The three plots on the left column are subset by time and samples are highlighted by color and shape based on oral locations (M; n = 51, A; n = 53, and E; n = 55). Vice versa, score plots on the right column are subset by location and highlighted by time (AT; n = 56, BT; n = 59, and CK; n = 44). Score plots display PC1 on the x axis and PC2 on the y axis.

**Figure 4:**
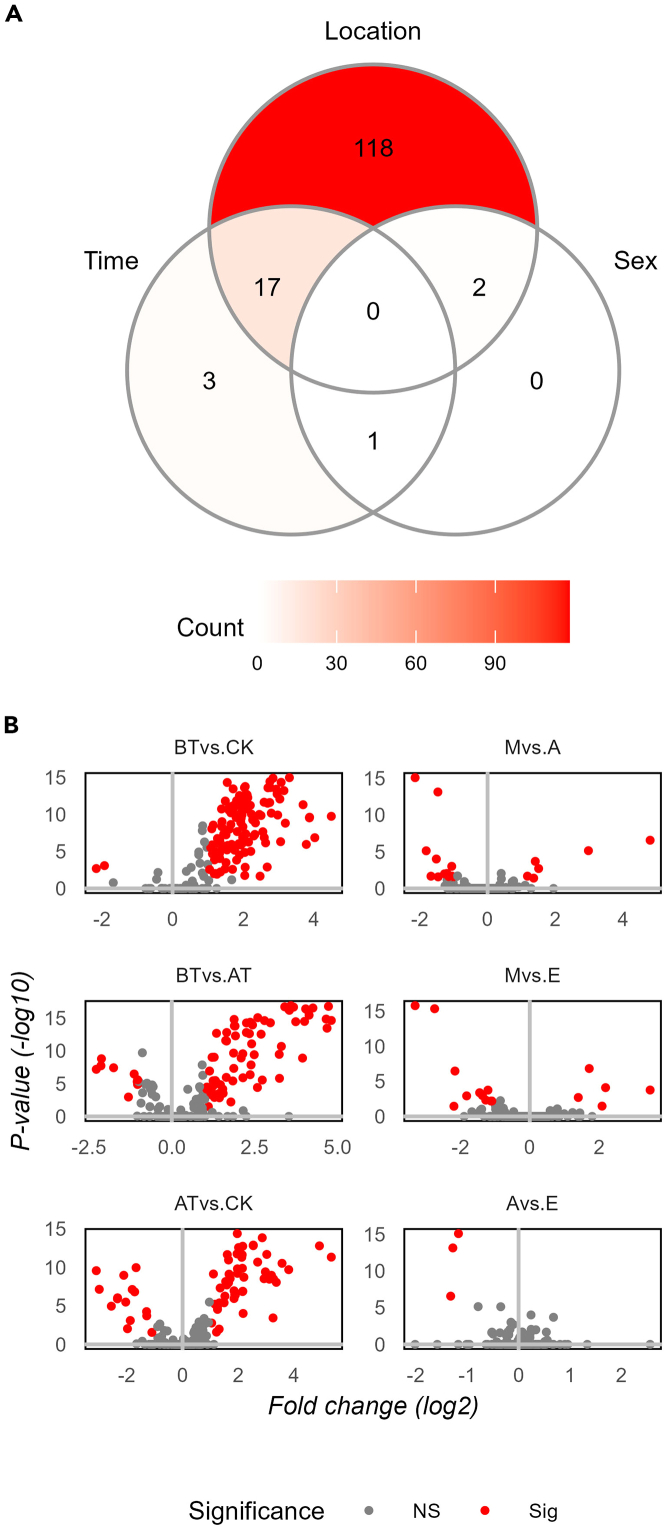
Statistical tests (A) The Venn diagram shows the significances and their overlap among categorical annotations (oral location, time of collection, and subject’s sex) by count. (B) Statistically significant metabolites are color coded in red in the volcano plot comparisons (adjusted p value <0.05 and fold change >2 or NS = non-significant) for three spatial comparisons (AT = above the tongue, BT = below the tongue, CK = cheek) and three time-based comparisons (M = morning, A = afternoon, E = evening). Volcano plots display Log_2_ of the fold change on the x axis and -Log_10_ of the p values on the y axis. P-values were computed using Wilcoxon Rank-Sum (AT; n = 56, BT; n = 59, CK; n = 44, M; n = 51, A; n = 53, and E; n = 55).

To temporally and spatially resolve the oral human metabolome, we analyzed 159 saliva samples from 20 healthy volunteers, including ten males and ten females (Table 1). Samples were collected at three different oral locations (spatially), above the tongue (AT), below the tongue (BT), and cheek (CK); as well as in a time-dependent fashion (temporally), morning (M), afternoon (A), and evening (E). To minimize the content of exogenous metabolites, donors were instructed to refrain from eating, smoking, brushing their teeth, and drinking, except plain water 1 h before collection. For a detailed description of the collection procedures please refer to the STAR Methods section. Using a semi-targeted LC-MS/MS platform based on an in-house metabolite library (see In-house LC-MS/MS library section for details), we identified a total of 177 metabolites, of which 135 are confirmed structures (level one) and 42 are tentative candidates (level three).^23^^,^^24^ Initially, we performed hierarchical clustering and Spearman correlation analysis. Heatmap analysis displayed mainly location-specific metabolic signatures (Figures 1 and S1). Subsequent correlation analysis showed a series of strong correlation clusters (Rho >0.8) (Figures 2 and S2). Many of the observed clusters are well known to be metabolically correlated, for instance: (1) the branched-chain amino acids (labeled as amino acids) cluster, including leucine, norleucine, and isoleucine, (2) the corticosteroids cluster (terpenoids), counting cortisol and cortisone, or (3) the caffeine cluster (alkaloids), where caffeine, paraxanthine, and theophylline (caffeine intermediates) are present among others. Subsequently, unsupervised Principal Component Analysis (PCA) was performed on temporal and spatial data subsets. PCA performed on spatial subsets display consistent separation of all three oral locations across all three time points of collection. Whereas, temporal subsets mainly displayed separation between morning samples and later time points at the BT location (Figure 3BT). No appreciable separation between time clusters was observed when AT and CK subsets were analyzed (Figures 3AT and 3CK). To further investigate these results, we carried out pairwise Wilcoxon rank sum tests revealing that 77% of the identified metabolites (137 out of 177) were significantly different between at least one pair of the oral cavity locations monitored (Bonferroni adjusted p value <0.05 and fold change >2), see Figure 4. Time-based comparisons identified 12% (21 metabolites) as significantly different (Figure 4). Furthermore, sex-based comparison displayed three significant metabolites; caffeine, paraxanthine, and theophylline (adjusted p value <0.05 and fold change >2), see Figure 4. All three metabolites belong to caffeine metabolism, which likely highlights the higher consumption of caffeine beverages by females in our study cohort rather than sexual dimorphisms. This significance vanished once location-based stratification was performed. Finally, no metabolite was correlated with the subject’s age or sleeping time. This is likely explained by the very narrow distribution of age (mean = 31.2, SD = 4.7) and sleeping time (mean = 7.5, SD = 0.7) within our cohort, (Table S1).

**Table 1:** Subject’s metadata

Subj.id	Sex	Age	Sleep (h)
A	Ma	30	6.5
B	Fe	31	8.25
C	Fe	29	7.75
D	Ma	38	8
E	Fe	29	8
F	Fe	31	7.75
G	Ma	24	7.25
H	Fe	28	7
I	Ma	27	8.25
J	Ma	41	7
K	Ma	30	8.75
L	Fe	29	8
M	Ma	31	8.25
N	Ma	28	NA
O	Ma	43	7.25
P	Fe	28	7.75
Q	Ma	30	7.5
R	Fe	31	6
S	Fe	36	6.5
T	Fe	30	7.25

Metadata includes sex, age, and time of sleep the night before sampling.

### Oral cavity location-specific metabolomes

**Figure 5:**
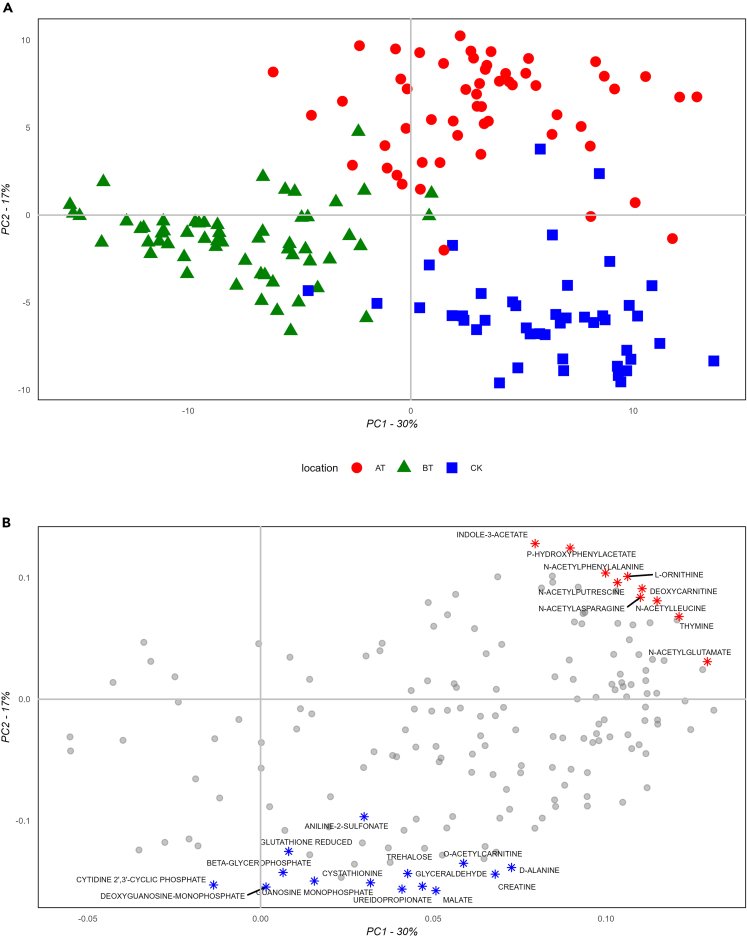
PLS-DA plots (A) Score plot displays PC1 on the x axis and PC2 on the y axis, samples are highlighted based on oral locations using colors and shapes (AT = above the tongue, BT = below the tongue, CK = cheek), and (B) Loading plot displays PC1 on the x axis and PC2 on the y axis, top VIPs (VIP >1.25) are highlighted by color and shape based on associated location and labeled with metabolite names (n = 159). Prior to PLS-DA computation, data were log-transformed, and *Z* score normalized. For diagnostic plots of the PLS-DA model see Figure S3.

A further refinement of the analysis was accomplished by Partial Least Squares Discriminant Analysis (PLS-DA) and Variable Importance for the Projection (VIP) coefficients. The PLS-DA score plot (Figures 5A and S3) showed three distinct clusters belonging to oral locations, which were in agreement with the PCA score plot (Figures 3M, 3A, and 3E). The most important metabolites for the projection (VIP >1.25), when highlighted on the loading plot, resulted in two specific clusters, as shown in Figure 5B. One cluster of metabolites was characteristic of AT (in red), whereas the second cluster (in blue) was distinctive of CK. In contrast, no VIP appeared to be unique to BT. Both the heatmap and PLS-DA plots suggested that BT was the least concentrated fluid among the three locations. Interestingly, AT was primarily characterized by N-acetylated amino acids and N-acetylated amino acid derivates, including N-alpha acetyl-lysine, N-acetyl glutamate, N-acetyl leucine, N-acetyl asparagine, N-acetyl phenylalanine, p-hydroxy-phenyl acetate, indole acetate, and N-acetyl putrescine. In addition, L-ornithine, thymine, and deoxy carnitine were also found to be characteristic of AT. On the other hand, CK was characterized by saccharides, counting glyceraldehyde, malate, trehalose, and sorbose, amino acids, and derivatives, i.e., alanine, ureidopropionate, and creatinine, sulfureted compounds, including reduced glutathione, cystathionine, and aniline-2-sulfonate, and, finally, mono-phosphorylated nucleotides i.e., guanosine monophosphate, deoxyguanosine monophosphate, and cytidine-2,3-cyclic phosphate. Metabolite distribution highlighted by PLS-DA was also visible in the correlation matrix (Figure 2), where N-acetylated metabolites, L-ornithine, thymine, and deoxy carnitine, as well as saccharides, sulfureted compounds, and mono-phosphorylated nucleotides clustered together. These spatial patterns suggest location-specific micro-environments. Location-specific read-outs could lead to new insights of gland-specific salivary metabolome functionalities, and spatially specific host-microbiome interactions.

### Salivary diurnal rhythms

**Figure 6:**
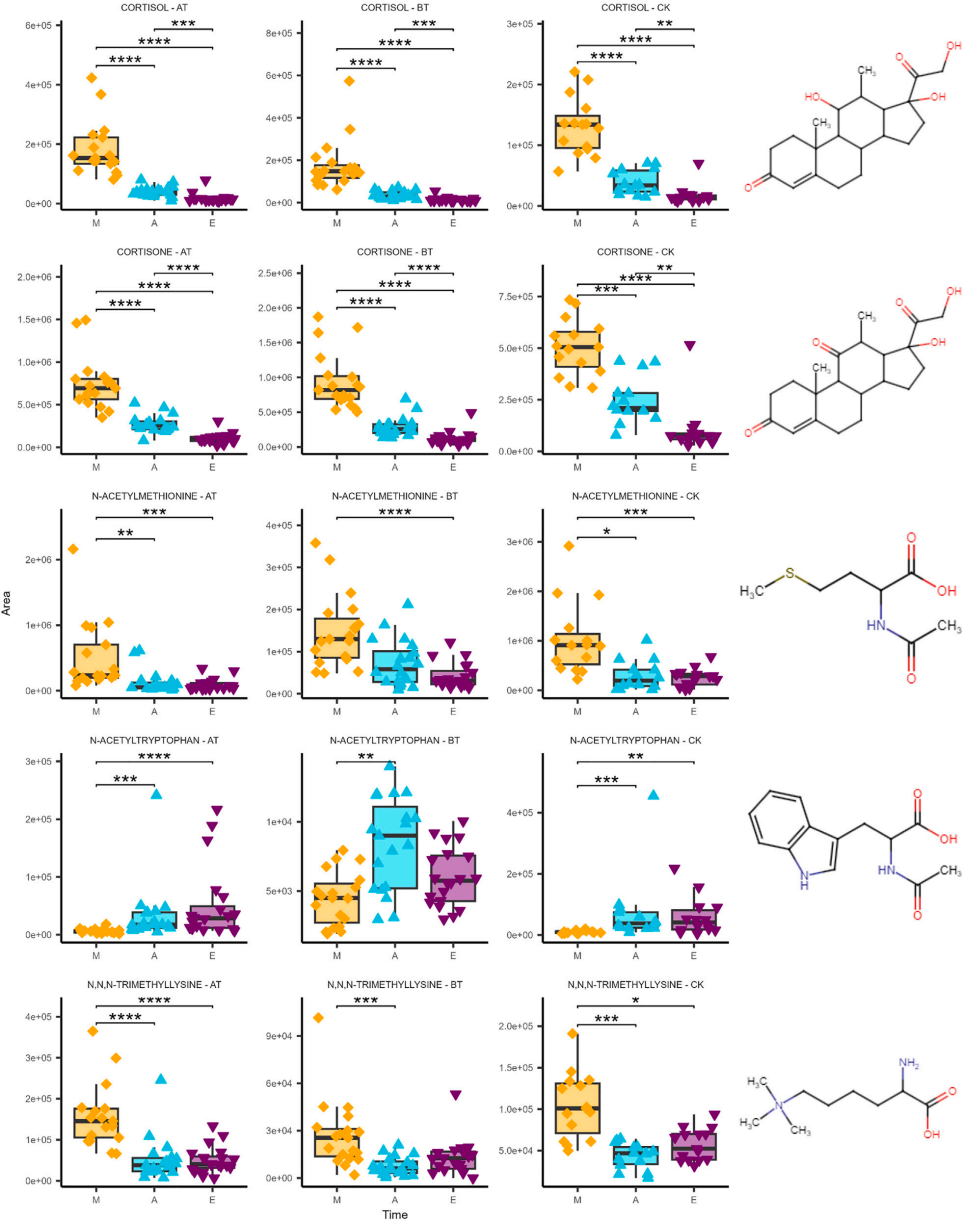
Top 5 time-dependent metabolites The boxplots above show metabolites by rows (From top to bottom, cortisol, cortisone, N-acetyl methionine, N-acetyl tryptophan, and N,N,N-trimethyl lysine) and stratified locations in columns (AT = above the tongue, BT = below the tongue, CK = cheek). For each plot, the x axis displays the time of collection and the y axis shows area intensity. Both boxplots and data points are highlighted for the time of collection (M = morning, A = afternoon, E = evening). Significances are calculated with the Wilcoxon rank-sum test and reported for all significant comparisons (∗∗adjusted p value <0.01, ∗∗∗adjusted p value <0.001, ∗∗∗∗adjusted p value <0.0001) (AT; n = 56, BT; n = 59, and CK; n = 44). The N-acetyl tryptophan boxplot excludes four extreme data points for visualization purposes. Error bars represent ± one standard deviation.

**Figure 7:**
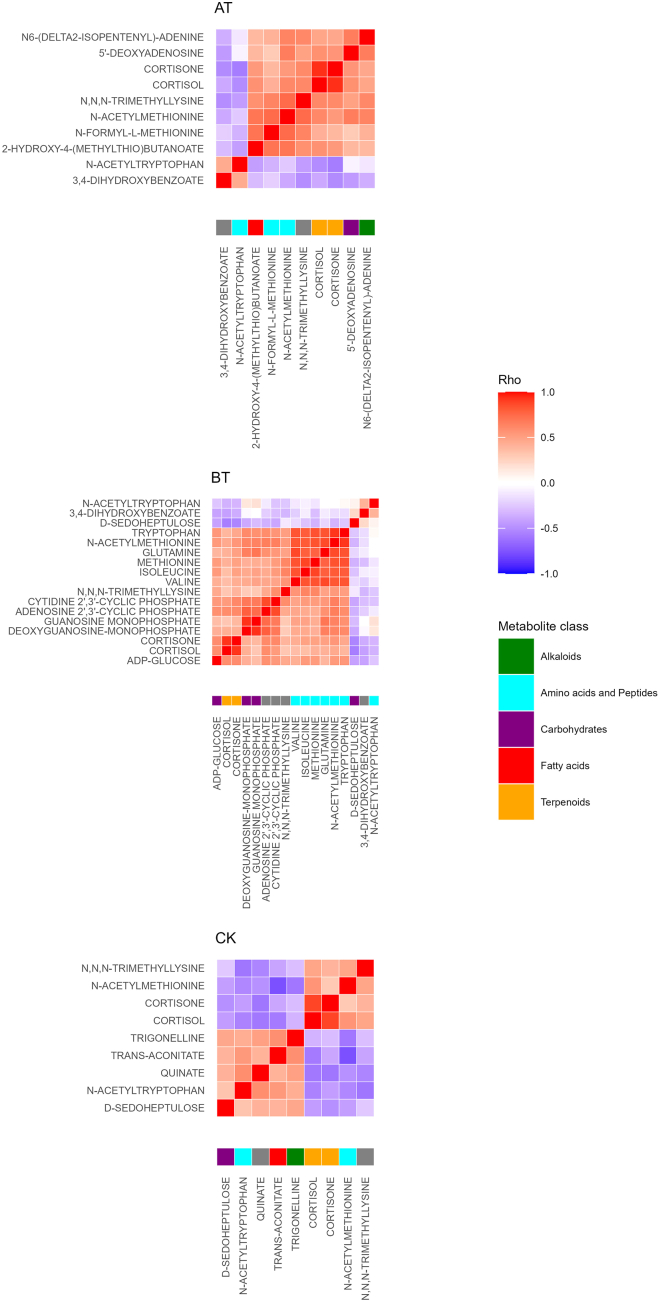
Spearman correlation matrices of time-significant metabolites stratified based on oral locations After the stratification of the data based on oral locations (AT = above the tongue, BT = below the tongue, CK = cheek) and computation of the time-based significances, correlation matrices were generated using Spearman correlation (AT; n = 56, BT; n = 59, and CK; n = 44). Metabolites are annotated with molecular classification on the pathway level as determined by Natural Product Classifier.^25^ Prior correlation data were log-transformed, and *Z* score normalized.

As a next step, we investigated longitudinal differences. The pairwise Wilcoxon Rank-Sum test on the time domain resulted in 21 metabolites displaying statistical significance (Bonferroni adjusted p value <0.05 and fold change >2), see Figure 4. Among the three time-based comparisons, M vs. A displayed the highest number of significant metabolites (#18) followed by M vs. E (#16), whereas A vs. E showed the least number of significantly different metabolites (#3) (Figure 4B). The significant metabolites in A vs. E; cortisol, cortisone, and N-acetyl tryptophan were consistently significant across all three time-comparisons. Moreover, the two corticosteroids and N-acetyl tryptophan proved to be the only metabolites significant in the time domain but independent from the spatial domain (Figure 4A). This does not hold true for the rest of the time-dependent metabolites, which were also location-dependent. Therefore, to investigate time-dependent metabolic patterns without spatial interference, we stratified our data based on locations before testing for differences in the time domain. When all three locations were separately investigated using pairwise testing, CK exhibited the least number of statistically significant metabolites (#9), followed by AT (#10), while BT displayed the highest number of significant metabolites (#17), see Figure 4B. These results were in agreement with PCA on subsets, where BT was the location displaying time-based clusters (Figure 3BT). Significant metabolites were defined as having an adjusted p value <0.05 and fold change >2. After spatial-stratification, five metabolites were statistically significant for the time domain across all locations: cortisol, cortisone, N-acetyl methionine, N,N,N-trimethyl lysine, and N-acetyl tryptophan (Figure 6). Interestingly, among these five metabolites, N-acetyl tryptophan showed a unique diurnal pattern marked by its nadir in the M and peaking later in the day. Moreover, once all time-based significant metabolites were superimposed (AT, BT, CK datasets), 71% (17 out of 24) were detectable in BT. Additionally, metabolites that displayed significance within the time domain were further analyzed using Spearman correlation. As expected, cortisol and cortisone showed a strong correlation in all locations (Rho = 0.94, 0.92, and 0.88, for AT, BT, and CK respectively, with p values <0.0001 for all comparisons). Corticosteroids were the only metabolites displaying a significant correlation in AT and CK (Figures 7AT and 7CK). On the other hand, BT displayed additional correlation clusters (Rho >0.8). The nucleotides adenosine 2′,3′-cyclic phosphate and cytidine 2′,3′-cyclic phosphate as well as deoxyguanosine monophosphate and guanosine monophosphate were correlated, and several amino acids were clustering together, including isoleucine, valine, methionine, N-acetyl methionine, taurine, tryptophan, and glutamate (Figure 7BT).

### Microbiome specific metabolites

Using the MiMeDB database^26^ we attempted to identify metabolites that were exclusively of microbial origin, but virtually all observed metabolites of interest can be produced both by microbes and humans. For instance, L-ornithine has been shown to be potentially important in the long-term colonization of specific gut pathogens such as Clostridioides difficile.^27^ However, in human metabolism ornithine is an important part of the urea cycle. Moreover, indole acetate is a metabolite resulting from tryptophan catabolism carried out by gut microbes,^28^ but it can be produced by human cells from both microbial and dietary tryptophan.^29^ In conclusion, because of the many facets a single metabolite can have across different metabolic pathways and ecosystems, it is not yet possible to discriminate metabolites based on their origin.

## Discussion

To the best of our knowledge, this is one of the first studies of its kind, where functional subtypes of saliva are spatially and temporally profiled. We identified 177 unique, metabolites within a single platform. Using simultaneous spatial- and time-resolved sampling, we shed light on the complexity of the oral human metabolome, dissecting it in multiple orthogonal dimensions. Particularly the spatial domain revealed remarkable differences between the oral locations.

### Oral location-specific metabolomes

Our results showed an uneven distribution of salivary metabolites throughout the oral cavity, with BT being the wateriest saliva subtype among the three sampled locations. Such a biochemical profile originated from site-specific intraoral flows, with BT (mandibular) being a site of high flow when compared with AT (maxillary)^1^ and accelerated clearance when compared with AT and CK locations.^30^ The high flow of saliva BT implies high clearance of exogenous components, microbiota-derived metabolites, and, consequently, a decreased overall complexity of BT saliva. The predominantly observed class of metabolites in AT were N-acetylated metabolites. Several oral health related studies have highlighted high levels of N-acetyl metabolites as a potential biomarker of a healthy oral cavity and low levels of oral inflammation. NMR-based studies have reported higher concentrations of acetylated small molecules in healthy individuals compared to patients with chronic periodontitis.^31^^,^^32^^,^^33^ Moreover, N-acetyl-leucine levels were found to be significantly lower in the saliva of patients with diabetic,^34^ and acetylated metabolite levels were proposed to discriminate between oral squamous cell carcinoma and oral lichen planus.^35^ To this end, the origin and the functional role of N-acetylated metabolites in the oral cavity are unknown. Additionally, AT was characterized by high levels of L-ornithine, which stimulates oxygen uptake by the microbiome and increases the oral pH.^36^ Increased oxygen uptake by bacteria has been suggested to promote the aerobic degradation of glucose and further oxidation of lactic acid into less harmful substances when referring to dental caries.^37^^,^^38^ In turn, L-ornithine could be a biomarker for healthy teeth. As an exception to the spatial patterns described above, N-acetyl aspartate and acetyl carnitine were found to be characteristic of CK rather than AT, whereas metabolically related metabolites, N-acetyl asparagine, and deoxy carnitine showed a reverse spatial distribution (Figure S4). These inconsistencies of spatial patterns raise questions about interplays between salivary gland-specific metabolites and location-specific host-microbiome interactions. It is well-known that spatial differences also exist in the oral microbiome, with the buccal mucosa (CK) microbiome differing in both microbial composition and function as compared to the tongue dorsum.^39^ While we cannot deduce the origin (human or microbial) of the metabolites in our study, it would be especially interesting to couple metabolome and microbiome profiles in a spatially and temporally resolved fashion. Surprisingly, four saccharides were distinctive for CK. As oral bacteria are known to ferment sugars into organic acids,^40^ a bacterial origin of these saccharides is unlikely. All four compounds might be of exogenous origin, e.g., food or oral hygiene products. However, the study subjects were instructed to collect morning samples after overnight fasting and avoiding any consumption of beverages except plain water. Therefore, if the origin of these compounds were exogenous, substantially higher amounts would have been observed in A and E samples rather than M. Yet, this was not the case. As a consequence, the release of saccharides from parotid glands is the most likely scenario. In particular, trehalose was suggested to protect membranes and proteins against damage and denaturation as a result of dehydration.^41^ Given the low salivary flow in CK (Dawes and MacPherson, 1993), the presence of saccharides could present an oral response mechanism of xerostomia. Finally, mono-phosphorylated nucleotides were found specifically in the CK, of which cyclic guanosine monophosphate levels are reported to consistently increase with the severity of olfactory pathologies.^42^

### Salivary diurnal rhythms

Among the sampling time points, M showed a distinctive profile, whereas A and E were the most identical, where the only significances were cortisol, cortisone, and N-acetyl tryptophan. After stratification, the Wilcoxon test identified five metabolites with significant temporal changes common to all three locations: cortisol, cortisone, N-acetyl methionine, N,N,N-trimethyl lysine, and N-acetyl tryptophan, see Figure 6. Cortisol and cortisone are the only metabolites that displayed statistical significance within the time domain without simultaneous significance in the spatial domain. This suggests that their secretion is not regulated by any specific salivary gland, but that these two metabolites are likely to derive from serum filtration. Indeed, cortisol is a well-known circadian biomarker that peaks after waking up and reaches its minimum at bedtime.^43^ In addition, cortisol levels in saliva paralleled serum cortisol levels across a 24-h period.^44^^,^^45^ Moreover, the plasma cortisone/cortisol ratio is reported to be constant during waking hours in healthy subjects^46^ and proved to be of clinical utility for assessing adrenal malfunction.^47^ Our data showed a high correlation between salivary cortisol and cortisone levels which could be a promising non-invasive measurement of adrenal function. Regarding other time-dependent metabolites, supplementation of methionine or tryptophan has been shown to affect activity/rest rhythm.^48^^,^^49^^,^^50^ However, the basis of the acetylated forms of these two metabolites and the circadian control of N,N,N-trimethyl lysine are unknown. Stratified data analysis highlighted BT as the oral location for most time-dependent metabolites. This may result from a specific circadian control over submandibular and sublingual glands, which contribute to the majority of saliva released in BT. Also, the high flow and high clearance of BT could clear exogenous metabolites and, consequently, enrich endogenous metabolites. In addition, when Spearman correlation analysis was performed separately on the three locations, only BT displayed correlations (Rho >0.8, p value = <0.01) other than cortisol and cortisone. Among these clusters, we observed nucleotides, including adenosine 2′,3′-cyclic phosphate and cytidine 2′,3′-cyclic phosphate, and amino acids, including valine, isoleucine, glutamine, methionine, N-acetylmethionine, and tryptophan (Figure 7). The former group of metabolites is known to derive from RNA degradation^51^^,^^52^ and hence may reflect salivary RNA transcription and, potentially, salivary protein production in response to circadian regulation. For the latter, the diurnal rhythm of amino acids in response to food intake has been described.^53^^,^^54^ However, our study was designed to minimize the influence of food intake by avoiding food consumption 3 h before saliva collection. Moreover, amino acid levels progressively decline from M (overnight fasting) to E, clearly pinpointing to circadian control. Interestingly, tryptophan and N-acetyl tryptophan showed no dependency by correlation analysis. This discrepancy might be explained by the fact that two metabolic pathways of tryptophan are under the control of two distinct circadian clocks: melatonin biogenesis which is regulated by the light/dark cycle and the kynurenine pathway which shows diurnal rhythmicity due to tryptophan pyrrolase which appears to be driven by adrenal rhythmicity.^55^

### Conclusions

Although we were unable to pinpoint microbiome specific metabolites, our spatial collection can provide the base for future multi-omics studies that combine and correlate microbial and metabolite sampling. The current study is one of the very few that have investigated the spatial distribution of the human oral metabolome.^9^ While Meleti et al. sampled only two oral locations and focused on major gland secretion, our study sampled at three locations, the cheeks as well as above and below the tongue, where only minor salivary glands contribute to the location-specific salivary secretions and we found remarkable differences. This result is a proof of concept that minor gland secretions can greatly contribute to oral metabolome heterogeneity and functionality. Another strength of our study is the use of a semi-targeted LC-MS platform allowing for expanded metabolic coverage when compared to NMR-based metabolomics as applied by Meleti et al.^9^ Such a wider view of the oral metabolome is a major advantage for a comprehensive understanding of oral physiology. Regarding temporal investigations, the majority of circadian investigations of the oral metabolome have been carried out in a targeted fashion focusing on a small number of metabolites. When considering untargeted investigations, as carried out by Dallmann et al.^21^ and Sugimoto et al.,^56^ our work adds an additional layer of information by accounting for location-specific circadian changes that have not been previously reported. We present a spatially and temporally resolved roadmap of the human oral metabolome. The spatial domain is the main discriminator of the oral metabolome, underlining its spatial heterogeneity and the distinct profile of salivary secretions. Among the metabolites that exhibit heterogeneous distribution, several important metabolites are linked to oral health, such as N-acetylated metabolites, L-ornithine, saccharides, and guanosine monophosphate. Next to the spatial distribution of metabolites, we identified known and novel time dependencies. Among the novel diurnal metabolites, N-acetyl methionine, N,N,N-trimethyl lysine, and N-acetyl tryptophan display diurnal patterns in all oral locations. The former two behave similarly to cortisol, and the latter has its nadir in the M. Moreover, a pool of amino acids and mono-phosphorylated nucleotides manifested a significant diurnal rhythm exclusively in BT. Taken together our findings, simultaneously, pinpoint limitations when analyzing this bodily fluid as a whole, and underscore the implications of spatial metabolomic analyses for future works. Particularly for oral disease (e.g., periodontitis or head and neck cancer) and biology-focused studies (e.g., tooth mineralization), spatial information is critical to reach accurate conclusions. Moreover, the effect of circadian rhythm on saliva composition is manifested unevenly within the oral cavity, and the use of whole saliva is likely to result in a dilution of diurnal alterations. In this regard, we suggest prioritizing BT as the preferred sampling location for the detection and monitoring of diurnal metabolic changes. In summary, we present a showcase pointing out the large heterogeneity of the oral environment. In this regard, spatial and temporal analysis of saliva could contribute to decoding the information hidden in this complex biofluid. Finally, as far as we are aware, spatial microbial investigations of the oral cavity have not yet been combined with metabolic analysis and would be crucial to advancing the field.

### Limitations of the study

The current investigation is focused on exploring the functional subtypes of saliva. The study design, however, does not allow to identify the precise origin of the observed metabolites. Although the implemented sampling schedule enabled the detection of diurnal changes, the restricted frequency of sample collection hinders the determination of the kinetics involved in these diurnal alterations. Moreover, it is important to note that essential information, such as the use of contraceptives and the phase of the menstrual cycle in female subjects, was not recorded. This omission limits the discriminatory power in gender comparisons. Another limitation is that our study design focused on healthy young male and female participants and no claims about potential temporal or spatial (patho-)physiological metabolic changes can be made. Moreover, we focused on the roughly 400 metabolites included in our in-house library and have not further investigated the obtained LC-MS data. Nevertheless, our data is publicly available and interested researchers can dive deeper into yet undiscovered spatial and temporal metabolic traits.

## STAR★Methods

### Key resources table

**Table:** 

REAGENT or RESOURCE	SOURCE	IDENTIFIER
**Chemicals, peptides, and recombinant proteins**		

Methanol LC-MS grade	Merck	1.06035.2500; CAS: 67-56-1
Water LC-MS grade	Honeywell	14263-2L; CAS: 7732-18-5
Formic acid additive for LC-MS	Honeywell	56302-10X1ML; CAS: 64-18-6

**Critical commercial assays**		

Swab Storage Tube	Salimetrics	https://salimetrics.com
Sugi® absorbent swab	Questalpha	https://www.questalpha.com

**Deposited data**		

Raw data, pos. ion. mode, batch-1	This paper	Mendeley Data, V1, https://doi.org/10.17632/fjkwnhmdjp.1
Raw data, pos. ion. mode, batch-2	This paper	Mendeley Data, V1, https://doi.org/10.17632/3ybszwwfww.1
Raw data, neg. ion. mode, batch-1	This paper	Mendeley Data, V1, https://doi.org/10.17632/tnbksjfv36.1
Raw data, neg. ion. mode, batch-2	This paper	Mendeley Data, V1, https://doi.org/10.17632/9v5bw5zr9j.1

**Software and algorithms**		

MS-DIAL (version 4.20)	Tsugawa et al., 2015	http://prime.psc.riken.jp/compms/msdial/main.html
R (version 4.0.3)	R Core Team, 2020	https://cran.r-project.org/

**Other**		

Synergi Hydro-RP LC column (100Å, 2.5 μm, 2 mm × 100 mm)	Phenomenex	00D-4387-B0
SecurityGuard ULTRA cartridges UHPLC C8 (2.1 mm)	Phenomenex	AJ0-8784

### Resource availability

#### Lead contact

Further information and requests for resources should be directed to and will be fulfilled by the lead contact, M. Giera (m.a.giera@lumc.nl).

#### Materials availability

This study did not generate new unique reagents.

#### Data and code availability

This study did not generate any code, all codes used are cited.

The data supporting the current study have been deposited at Mendeley Data and are publicly available as of the date of publication. Accession numbers are listed in the key resources table.

Any additional information required to reanalyze the data reported in this paper is available from the lead contact upon request.

### Experimental model and study participant details

This study was conducted according to the Declaration of Helsinki principles.^57^ The study protocol for “*spatially- and temporally-resolved biochemical profiling of saliva*” was approved by the CCB science committee and the Medical Ethical Committee of the LUMC (METC-LDD). Study volunteers were excluded from the study if: (1) they worked night shifts, (2) took any medication, or (3) had any suspected/known medical condition with special attention to oral disease, e.g. periodontitis. Therefore. all study subjects, ten males and ten females, were healthy volunteers between the ages of 18 and 45 years old (Table 1). Saliva samples and demographic information were encoded (pseudonymized). Samples were collected three times a day; one hour after waking up - Morning (M), three hours after lunch - Afternoon (A), and one hour before bedtime - Evening (E) on three different oral locations; above the tongue (AT), below the tongue (BT), and right cheek (CK). Saliva collected from the right cheek was considered representative of both cheeks, based on previous research, which showed no significant differences between the left and right cheek using identical spatially-resolved collection procedures.^8^ Due to the necessary sampling time for the detection of circadian-related metabolites, donors were responsible for collecting their saliva using a collection kit provided by the research team. Detailed documentation of the procedure was included in the kit and volunteers were instructed on procedures and precautions before sample collection. Donors were recommended to drink half a liter of water between three hours to one hour before saliva collection to facilitate collection and had to refrain from eating, smoking, drinking (except water), and brushing their teeth one hour before collection to minimize any exogenous metabolites in the collected saliva samples. That being the case, morning samples were collected after overnight fasting, without beverage consumption (except water). Moreover, donors were also requested to rinse their mouths with plain water ten minutes before collection. Spatially resolved saliva was collected using the Eyespear swabs by placing a swab on an oral area of interest for a duration of two and a half minutes before storage in a swab storage tube. Volunteers were requested to store saliva samples in the freezer (-20°C) after collection and to deliver them using a polyester box and ice bag provided with the collection kit. All boxes received contained solid ice and no defrosting had occurred. Upon receipt, the samples were centrifuged at 1500 ×g for 15 min at 4°C and the volume of collected saliva per swab was assessed. Samples with less than the minimum volume required for the analysis (< 40 μL) were discarded, while all other samples were stored at -80°C until analysis. due to low saliva volume, 21 out of 180 samples were discarded. For a step-by-step guide about how to perform spatially resolved sampling of saliva and a detailed description of the oral anatomical boundaries used in this study, see.^58^ Additionally, the following anonymized data were collected: sex, age, and sleeping time before collection (Table 1). After a careful statistical analysis, demographic descriptors did not display any significant influence in our dataset. Finally, no information related to ancestry, race, or ethnicity was collected.

### Method details

#### Materials

Methanol was purchased from Merck (Darmstadt, Germany). If not stated otherwise, all chemicals were purchased from Honeywell (Charlotte, NC, USA). All solvents were of LC-MS grade. Eyespears were from Sugi® products (Questalpha). Swab storage tubes were obtained from Salimetrics.

#### Sample preparation

After overnight thawing at 4°C, an aliquot of 40 μL from each sample was transferred to a 1.5 mL Eppendorf tube and 160 μL of ice-cold MeOH was added. Subsequently, samples were placed in a freezer at -20°C for 20 min to complete protein precipitation. Next, samples were centrifuged at 18000 ×g for 20 min at 4°C and supernatants were transferred to 1.5 mL Eppendorf tubes. Samples were dried under a gentle stream of nitrogen and reconstituted in 40 μL of 1:99 MeOH:H_2_O (v/v %). Reconstitution was assisted by sonication for one minute and vortexing for 5 seconds. The samples were analyzed in both ESI+ and ESI- modes.

#### Instrumental parameters

Chromatography was performed using a Nexera X2 system (Shimadzu, Duisburg, Germany) equipped with a Synergi Hydro-RP LC column (100Å, 1.7 μm, 2 mm × 100 mm) (Phenomenex, Aschaffenburg, Germany) that was kept at 40°C. The injection volume was 10 μL and gradient elution was accomplished using H_2_O with 0.1% formic acid (eluent A), and MeOH with 0.1% formic acid (eluent B). The flow rate was 0.4 mL min^-1^. The gradient was as follows: 0 to 1.5 min - 0% B, linearly increased to 97% B at 9.9 min, 9.9 to 12.9 min - 97% B, and 13 to 13.8 min - 0% B. For detection, a Sciex TripleTOF 6600 Q-TOF mass spectrometer (Sciex, Framingham, MS, USA) was used, scanning from *m/z* 75 to 650 in both positive (ESI+) and negative (ESI-) electrospray ionization modes. A detailed listing of all settings can be found in see below table.

**Table:** MS settings

**MS Instrument**	

Instrument type	Q-TOF instrument (TripleTOF 6600, Sciex)

**Source**	

Source Type	ESI (Turbo V Ion Source, Sciex)
CUR gas	30
GAS 1	50
GAS 2	50
ISVF	5500 V (−4500 V)
TEM	500°C

**TOF MS**	

Duration	13.801 min
Cycles	974
Cycle time	0.8502 s
DP	80 eV (−80 eV)
CE	10 eV (−10 eV)
Start mass	75 Da
End mass	650 Da
Accumulation time	0.079985 s

**Product Ion**	

Acquisition method	IDA
With intensity greater than	100 cps
Maximum number of candidates to monitor per cycle	18
Exclude former target ions	Never
Mass tolerance	50 ppm
DP	80 eV (−80 eV)
CE	30 eV (−30 eV)
CES	15 eV (−15 eV)
Start mass	35 Da
End mass	650 Da
Accumulation time	0.040012 ms

MS settings required to ensure reproducible MS measurements are here reported, related to STAR Methods. When parameters diverge between ESI+ mode and ESI- mode, ESI- parameters are reported in brackets.

#### Quality control and batch structure

For quality control purposes, blank samples (water) and QC pool samples (pool of all study samples) were included in the analysis. The batch structure involved an equilibration sequence of eight injections comprising four blanks and four QC pools, before starting sample analysis. The general batch structure was as follows, one QCpool, five samples, one QCpool, and one blank. All samples were randomized before analysis. Instrument calibration was performed with intervals of eight injections using CDS (Sciex, Framingham, MS, USA).

#### Data pre-processing

Spectra deconvolution, peak alignment, and compound identification were performed using MS-DIAL version 4.90.^59^ A full list of all applied parameters can be found in see below table. Metabolite identification was achieved by matching exact precursor mass, retention time, adduct formation, and fragmentation spectra with an in-house LC-MS/MS spectral library of authentic compounds (see below) analyzed under identical experimental conditions. Based on consensus within the metabolomic field,^23^^,^^24^ metabolites were reported as validated (level one) when features were matched for exact mass, RT, and MS/MS spectra using our in-house library or tentative structure (level three) when the library match is achieved only for exact mass and RT without MS/MS spectra matching. Identification confidence levels are reported per metabolite in Table S1. After the exclusion of unknowns, MSDIAL outcomes showed 200 and 152 features provided with an identity in ESI+ and ESI- mode respectively. Subsequently, data were filtered for missing values, and relative standard deviation using the QCpool (RSD). For the former, metabolites with signals lower than the blank average in more than 66% of the samples were removed, and 9 and 5 compounds were excluded from ESI+ and ESI- mode respectively. For the latter, compounds with RSD higher than 30% as obtained in QCpool samples were excluded, 80 and 22 metabolites were removed for ESI+ and ESI- mode respectively. In addition, all adducts belonging to the same metabolite were summed up, and for metabolites identified in both ionization modes, the ion form with the higher RSD was excluded. After the adducts sum, 100 and 121 unique metabolites were obtained for ESI+ and ESI- mode respectively, and after merging the two datasets 177 unique and structurally identified metabolites were assembled in a single dataset and used for further analysis.

**Table:** MS-DIAL settings

MS-DIAL Version	4.80
Project	
MS1 Data type	Profile
MS2 Data type	Profile
Ion mode	Positive (Negative)
Target	Metabolomics
Mode	ddMSMS

**Data collection parameters**	

Retention time begin	0.5
Retention time end	12
Mass range begin	75
Mass range end	650
MS2 mass range begin	30
MS2 mass range end	650

**Centroid parameters**	

MS1 tolerance	0.01
MS2 tolerance	0.025
Isotope recognition and data processing	
Maximum charged number	2
Number of threads	8
Peak spotting parameters	
Mass slice width	0.1

**Peak detection parameters**	

Smoothing method Linear	LinearWeighted Moving Average
Smoothing level	2
Minimum peak width	5
Minimum peak height	500 (300)

**Deconvolution parameters**	

Sigma window value	0.5
MS2Dec amplitude cut off	1
Exclude after precursor	True
Keep isotope until	0.5
Keep original precursor isotopes	False

**MSP file and MS/MS identification setting**	

MSP file	In-house library
Retention time tolerance	0.3
Accurate mass tolerance (MS1)	0.01
Accurate mass tolerance (MS2)	0.05
Identification score cut off (%)	70
Using retention time for scoring	True
Using retention time for filtering	True

**Text file and post-identification (retention time and accurate mass-based) setting**	

Retention time tolerance	0.1
Accurate mass tolerance	0.01
Identification score cut off	85

**Advanced setting for identification**	

Relative abundance cut off	0
Top candidate report	False

**Adduct ion setting**	

[M+H]+ ([M-H]-)	True
[M+NH4]+ ([M+FA-H]-)	True
[M+Na]+ ([M+Cl]-)	True
[M+K]+	True
[M+H-H2O]+ ([M-H2O-H]-)	True
[M+2H]2+ ([M-2H]2-)	True

**Alignment parameters setting**	

Retention time tolerance	0.3
MS1 tolerance	0.02
Retention time factor	0.5
MS1 factor	0.5
Peak count filter	0
N% detected in at least one group	100
Remove feature based on peak height fold-change	True
Sample max/blank average	False
Sample average/blank average	2
Keep identified and annotated metabolites	False
Keep removable features and assign the tag for checking	False
Gap filling by compulsion	True

**Tracking of isotope labels**	

Tracking of isotopic labels	False

**Ion mobility**	

Ion mobility data	False

MS-DIAL settings used for detection, deconvolution, alignment, and identification are here reported, related to STAR Methods. When parameters diverged between ESI+ mode and ESI- mode, ESI- parameters were reported in brackets.

#### In-house LC-MS/MS library

Using the Sigma Aldrich Mass Spectrometry Metabolites Library of Standards (MSMLS) kit, we created an in-house database for both negative and positive ionization modes. The kit comprises 603 highly pure small molecules (>95%). For details of the MSMLS kit see the product specification (sigmaaldrich.com). To build the library, the Synergi Hydro-RP column (Phenomenex) was employed, as it demonstrated the most reproducible and stable results during preliminary tests (data not shown). For the complete list of LC and MS conditions, refer to the instrumental parameters section and Table 2, respectively. Before first use, all plates were centrifuged for 20 minutes at 4000 rpm and 4°C. The authentic standards were resuspended in accordance with the manufacturer’s guidelines. Authentic compounds were measured in mixtures of 12 standards, with each sorted by molecular weight, each standard mix was injected in triplicates at three working concentrations (1 μg/mL, 0.5 μg/mL, and 0.25 μg/mL). Hydrophobic standards (Log p < 1) were resuspended in MeOH:H_2_O 5:95 v/v, whereas hydrophilic standards (Log p >= 1), were resuspended in MeOH:H_2_O 1:99 v/v. The use of multiple concentrations enabled to distinguish between system- or analyte-related peaks as well as avoided problems related to detection and saturation limits for each compound. Whereas, injecting in triplicates provided a reasonable number of replicates to calculate RT average and RT deviation (n=9), as well as MS/MS consensus spectra, for which only the highest concentration was used (n=3). The LC-MS/MS data obtained were converted to “.mzXML” files using Msconvert, with parameters specified in see below table. Next, “.mzXML” files were imported into R Studio for data processing. Using R, peak detection was carried out using the CentWave algorithm^60^ and metabolites were annotated when peaks were detected in all replicates. For each metabolite the following parameters were calculated and reported: RT mean, RT deviation (RTmax – RTmin), and consensus MS/MS spectra. The consensus spectra provided a list of fragments detected in all of the 1 μg/mL replicates, where intensities were reported as the mean and, subsequently converted into a percentage referring to the mass with the highest intensity set at 100%. Ultimately, 406 unique metabolites were characterized and RT, adduct formation, and MS/MS spectra (312 positive and 302 negative ionization modes, respectively) were recorded.

**Table:** MSconvert settings

MSconvert Version	v3.0.19346
Output format	mzXML
Binary encoding precision	64-bit
Write index	Checked
Use zlib compression	Checked
TPP compatibility	Checked
Peak Picking	Vender (MS Levels 1 – NA)

Note: Peak picking MUST be the first filter applied.

MSconvert settings used for data conversion are here reported, related to STAR Methods.

### Quantification and statistical analysis

To explore our dataset both spatially and temporally, a hierarchical clustering dendrogram including all available categorical annotations was utilized to display the relationships among the samples and their major contributors. Before heatmap analysis, data were log-transformed and z-score normalized. Clusters were obtained using the Ward2 algorithm.^61^ In addition, to shed light on the metabolic network of the oral cavity, Spearman’s ranks correlation coefficients were calculated to identify correlated metabolites. Subsequently, PCA analysis on subsets was performed to reveal additional contributors. The study dataset was subset using location and time of collection. Before PCA computation, data were log-transformed and z-score normalized. Moreover, pairwise Wilcoxon rank sum test was applied for oral locations (AT vs. BT, AT vs. CK, and BT vs. CK), time of collection (M vs. A, M vs. E, and A vs. E), and sex (Male vs. Female). Due to the multiple statistical tests being performed on the dataset (n = 177), p-values were adjusted using Bonferroni correction. Moreover, fold changes were calculated for all comparisons. Overlap between significant annotations was displayed with a Venn diagram, whereas the distribution of significant metabolites was carried out using volcano plots. P-values and fold changes for all comparisons are reported in Table S1. Oral location-specific metabolites were identified using PLS-DA and Variable Importance in Projection (VIP) scores with the aim of clustering and ranking metabolites that discriminate between oral locations. Metabolites with a VIP score above 1.25 were further discussed in the present manuscript. Before PLS-DA computation, data were log-transformed and z-score normalized. The PLS-DA model was validated through a permutation test, model parameters (R2Y and Q2), and observational diagnostics, see Figure S2. VIP values for all metabolites are reported in Table S1. In contrast, time and sex were further investigated after location-based stratification. While, after stratification, sex did not display any significance, time was further investigated using a Wilcoxon test, and correlation analysis with each location subset. Data preprocessing was applied in the same fashion as described for the full dataset. All p-values and fold changes for stratified datasets are reported in Tables S2, S3, and S4 for AT, BT, and CK respectively. Data analyses and visualizations were carried out using R (R version 4.3.0).^62^ The packages used during data analysis were Tidyverse,^63^ ggforce,^64^ Scales,^65^ Patchwork,^66^ ropls,^67^ pheatmap,^68^ ggVennDiagram,^69^ and corrplot,^70^ ggpubr,^71^ rstatix.^72^ Classification of metabolites was accomplished using the following procedure: chemical structures obtained from the in-house database were converted from SMILES to InChIKeys using rdKit. Classifications were then retrieved for each compound via their InChIKeys with ClassyFire and the ClassyFireR package^73^ for Table S5 showing the complete data set. For Figures 1, 2, 7, and S1 classification was accomplished using NP Classifier,^25^ with the chemodiv package.^74^ The complete set of classifications can be found in Table S5.

## Supplemental information

Document S1. Figures S1–S4

Table S1. List of all detected metabolites, related to Figures 1, 3, and 5 All detected metabolites are reported for the level of identification, p-values, fold changes, and VIP scores relative to the PLS-DA model.

Table S2. P-values and fold changes for the AT-stratified dataset, related to Figure 7AT

Table S3. P-values and fold changes for the BT-stratified dataset, related to Figure 7BT

Table S4. P-values and fold changes for the CK-stratified dataset, related to Figure 7CK

Table S5. Classification of annotated metabolites, related to Figures 1, 2, 5, 7, and S1
