# Supplementary material for: Spatially and temporally resolved metabolome of the human oral cavity
Source: iScience. 2024 Jan 12;27(2):108884. doi: 10.1016/j.isci.2024.108884 (PMC10839270; doi:10.1016/j.isci.2024.108884)
Supplement: Document S1. Figures S1–S4 [file mmc1.pdf]

iScience, Volume *27*

Supplemental information

Spatially and temporally resolved

metabolome of the human oral cavity

Alessio Ciurli, Yassene Mohammed, Christine Ammon, Rico J.E. Derks, Damien Olivier-Jimenez, Quinten R. Ducarmon, Marije Slingerland, Jacques Neefjes, and Martin Giera

Supplemental Information

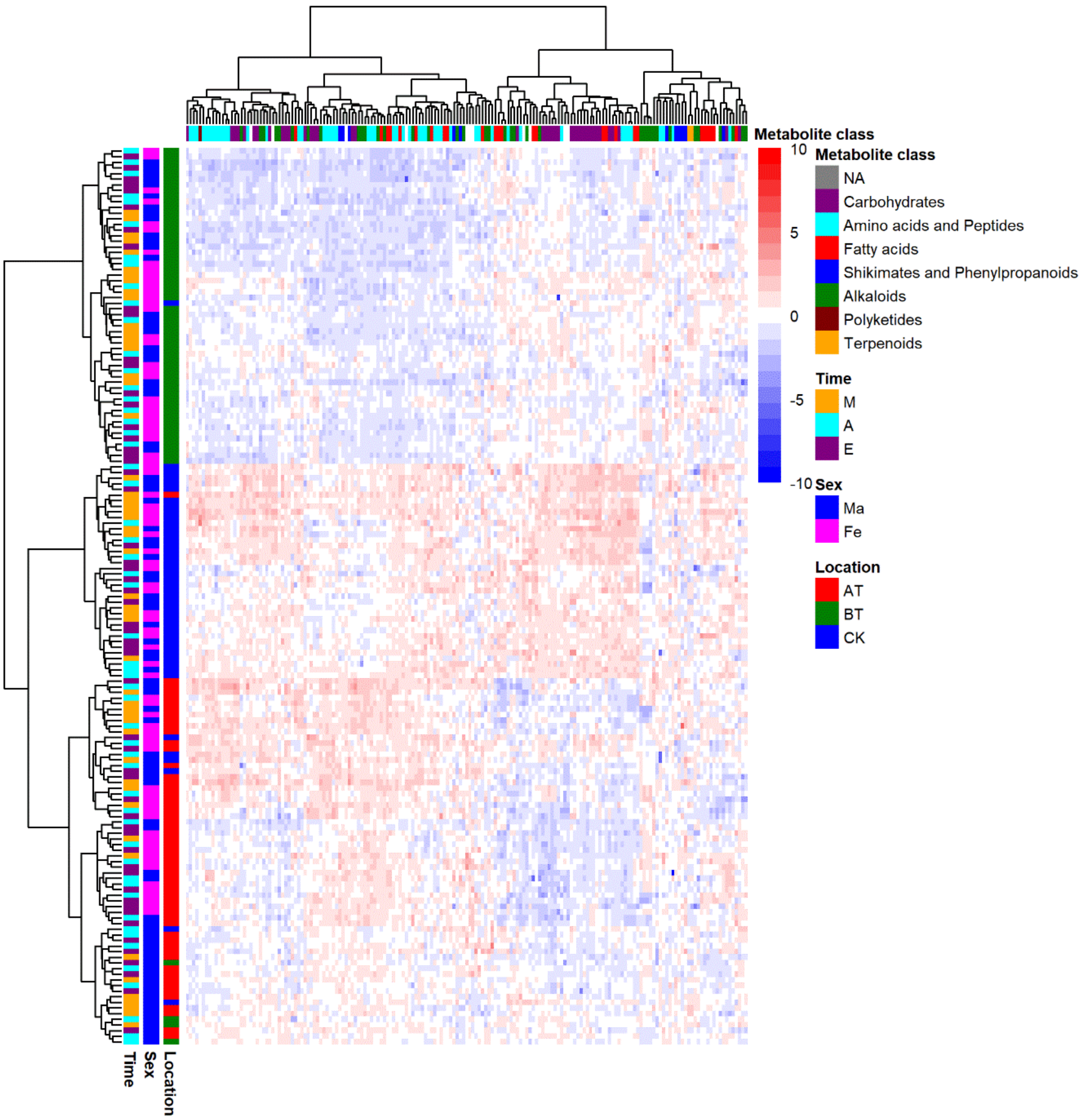

Figure S1. Heatmap of the complete salivary metabolic profiles, related to Figure 1

Rows are samples and columns are metabolites. Determined metabolite levels were scaled and centered. Samples are annotated by time (M = morning, A = afternoon, E = evening), location (AT = above the tongue, BT = below the tongue, CK = cheek), and sex (Ma = male, Fe = female). Metabolites are annotated with molecular classification on the pathway level as

determined by Natural Product Classifier[1]. For a full in-depth annotation using ClassyFire [2] please refer to Table S5.

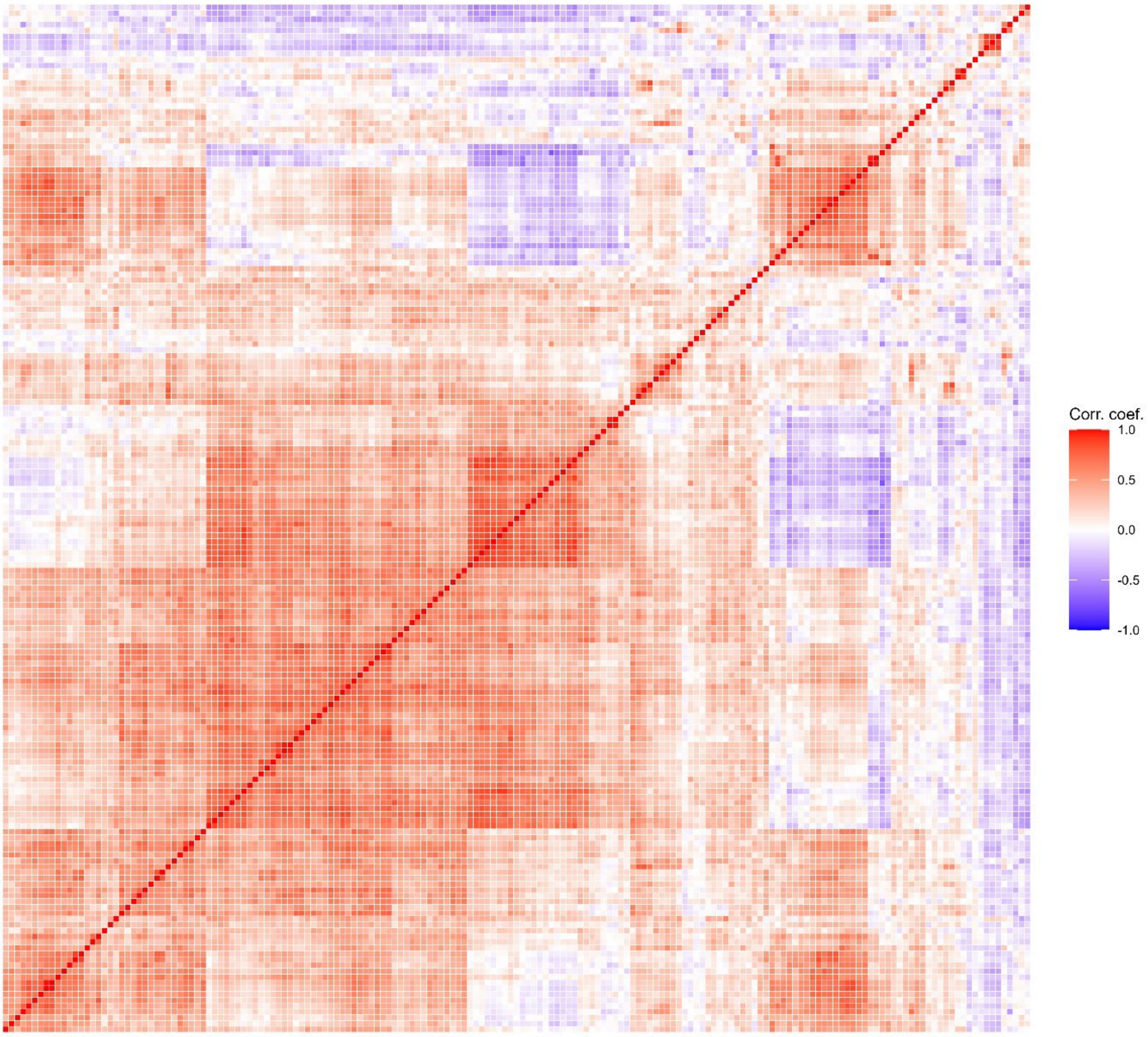

Figure S2. Spearman correlation matrix of all identified metabolites, related to Figure 2

The correlation matrix was generated using Spearman's rank correlation. Prior correlation, data were log-transformed, and z-score normalized.

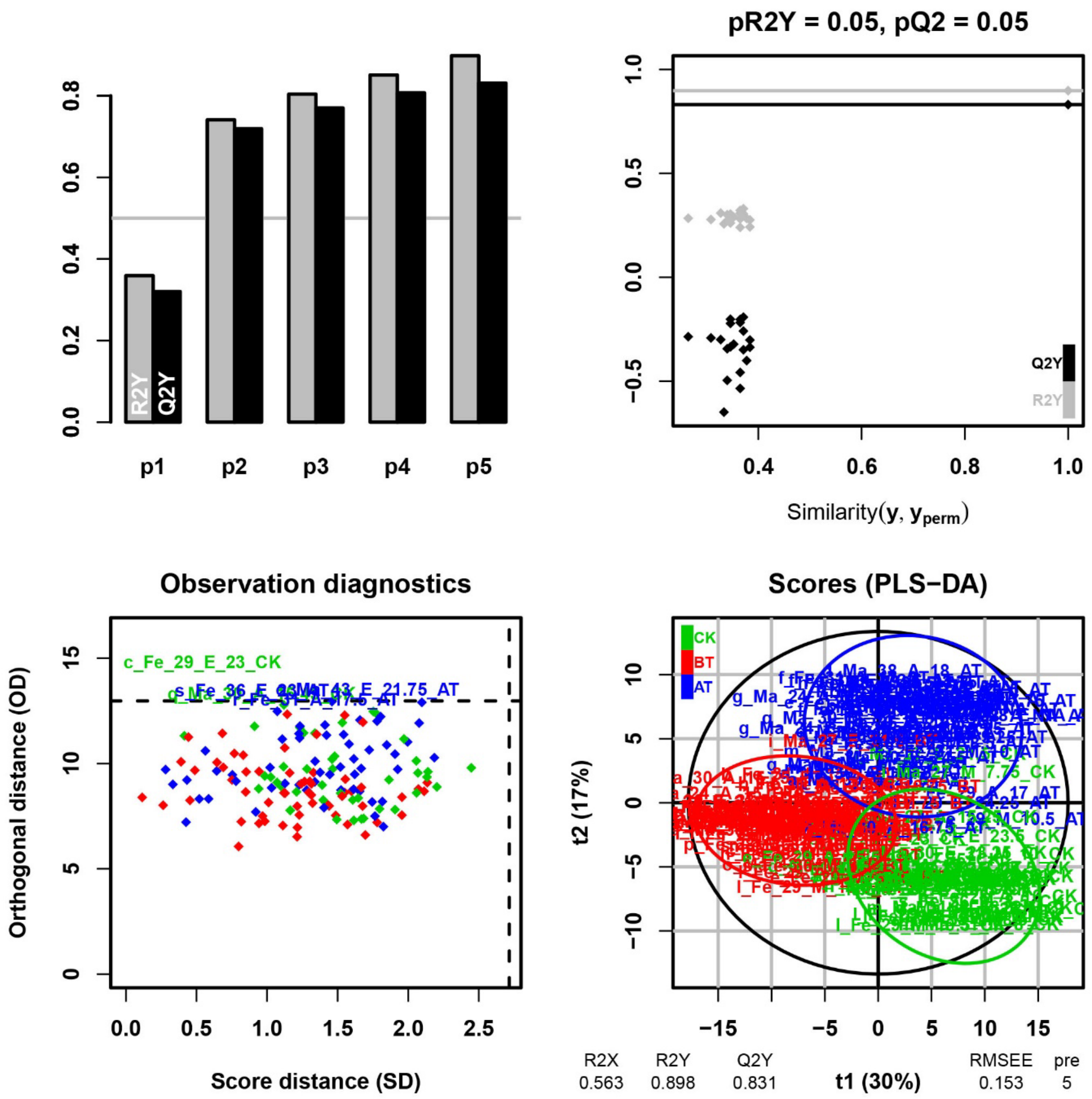

Figure S3. Diagnostic plots from ropls package, related to Figure 5

Top-left: model parameters barplot. Top-right: permutation test. Bottom-left: observation diagnostic. Bottom-right: scores plot.

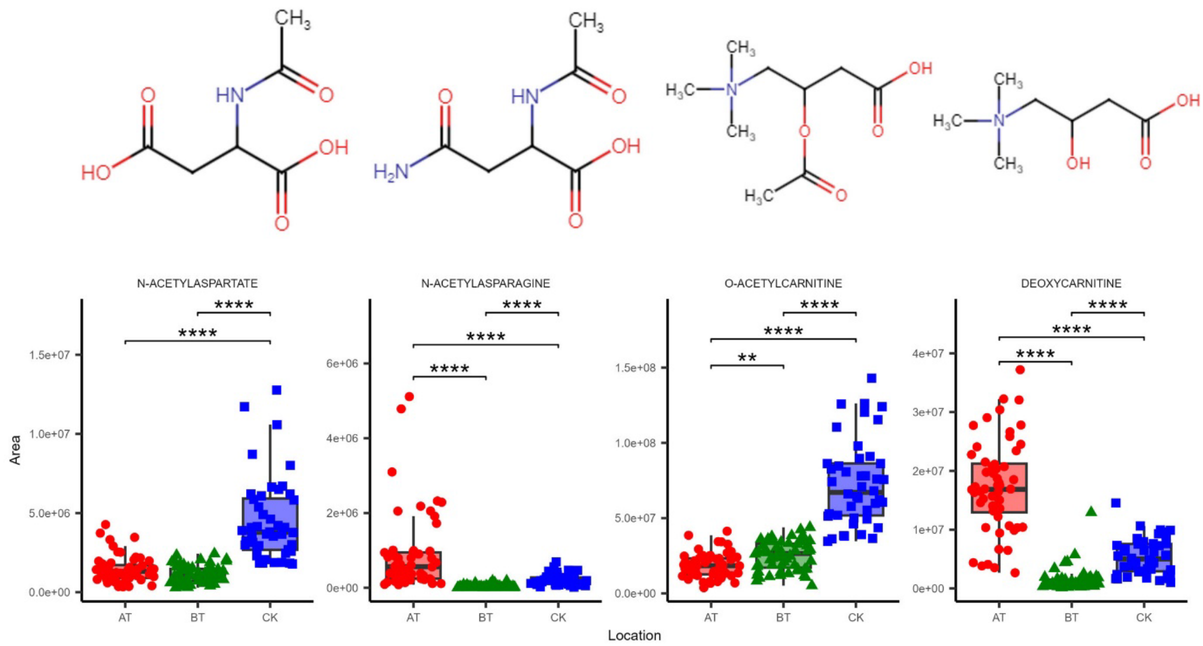

Figure S4. Location-based box plots, related to Figure 5

For each plot, the x-axis displays the oral locations and the y-axis shows area intensity. Both boxplots and data points are highlighted for the oral location of the collection. Chemical structures of location-dependent metabolites are reported on the right column accordingly. Significances are calculated with the Wilcoxon rank sum test and reported for all significant comparisons (**adjusted p-value <0.01, ***adjusted p-value <0.001, ****adjusted p-value <0.0001) (ATn = 56, BTn = 59, and CKn = 44).

Supplemental References

1. Kim, H.W., Wang, M., Leber, C.A., Nothias, L.-F., Reher, R., Kang, K.B., van der Hooft, J.J.J., Dorrestein, P.C., Gerwick, W.H., and Cottrell, G.W. (2021). NPClassifier: A Deep Neural Network-Based Structural Classification Tool for Natural Products. Journal of Natural Products *84*, 2795-2807. 10.1021/acs.jnatprod.1c00399.
2. Djoumbou Feunang, Y., Eisner, R., Knox, C., Chepelev, L., Hastings, J., Owen, G., Fahy, E., Steinbeck, C., Subramanian, S., Bolton, E., et al. (2016). ClassyFire: automated chemical classification with a comprehensive, computable taxonomy. Journal of Cheminformatics *8*, 61. 10.1186/s13321-016-0174-y.
